# Menstrual irregularities following COVID-19 vaccination: A global cross-sectional survey

**DOI:** 10.1016/j.amsu.2022.104220

**Published:** 2022-08-06

**Authors:** Azza Sarfraz, Zouina Sarfraz, Muzna Sarfraz, Zainab Nadeem, Miguel Felix, Ivan Cherrez-Ojeda

**Affiliations:** a The Aga Khan University, Karachi, Pakistan; b Fatima Jinnah Medical University, Lahore, Pakistan; c King Edward Medical University, Lahore, Pakistan; d Universidad Espíritu Santo, Samborondón, Ecuador; e Respiralab, Respiralab Research Group, Guayaquil, Ecuador

**Keywords:** Menstruation, COVID-19, SARS-CoV-2, Vaccination, Women's health

## Abstract

**Introduction:**

The coronavirus disease 2019 (COVID-19) vaccination generates protective immunity against SARS-CoV-2 infection. There is no clear evidence of COVID-19 vaccine-induced menstrual irregularities.

**Objective:**

To identify potential menstrual irregularities following COVID-19 vaccine among females.

**Methods:**

A worldwide cross-sectional survey study was conducted from June 10, 2021, to July 10, 2021 using online mediums. The survey consisted of 15 questions divided into baseline characteristics, vaccination status and dosage, menstruation and relate factors, and thoughts and knowledge about menstrual irregularities. Non-probability convenience sampling method was used including 510 responses. The results were tabulated, with bivariate analysis and chi-square test results. The sensitivity and specificity test of factors associated to knowledge about menstrual irregularities post COVID-19 vaccination were analyzed by receiver operating characteristic analysis.

**Results:**

The associations between healthcare worker (HCW) status and perceptions (χ2 = 10.422; p = 0.064), and knowledge about menstrual irregularities post-vaccination (χ2 = 1.966; p = 0.161) were found. Vaccinated compared to non-vaccinated women had a higher risk of change in inter-cycle length between periods (OR = 3.172; 95% CI = 0.470–21.431). Of 314 HCW vs. 196 non-HCW, 60 (19.1%) vs. 28 (14.3%) were knowledgeable about menstrual irregularities (OR = 1.338, 95% CI = 0.886–2.019 vs. OR = 0.944; 95% CI = 0.873–1.021). On asking the HCW vs. non-HCW about perceptions of COVID-19 vaccine-induced menstrual irregularities, 24 (7.6%) vs. 9 (4.6%) agreed, 139 (44.3%) vs. 67 (34.2%) disagreed, and 151 (48.1%) vs. 120 (61.2%) did not know or chose not applicable.

**Conclusion:**

There is a gap in the current understanding of menstrual irregularities, even if temporary, following COVID-19 vaccination that requires further exploration. Misinformation may also be the culprit for the observed proportion of women that noticed changes in their menstrual periods after COVID-19 vaccination.

## Highlights

• COVID-19 vaccines lead to protective immunity against SARS-CoV-2 infection.
• There is no clear evidence of COVID-19 vaccine-induced menstrual irregularities.
• Associations between healthcare worker status and perceptions, and knowledge about menstrual irregularities post-vaccination were noted.
• Misinformation may also be the culprit for noticed changes in menstrual periods after COVID-19 vaccination.

## 1 Introduction

The COVID-19 vaccination has been observed to generate protective immunity against SARS-CoV-2 infection with anecdotal evidence of menstrual irregularities possibly due to physical reaction to the vaccine. There is no clear evidence of COVID-19 vaccine-induced menstrual irregularities. Additionally, there are scientific associations of sex-specific responses to influenza vaccination with stronger humoral and cellular responses among women [1,2]. Declined ovarian reserve was observed in women of reproductive age with COVID-19 infection as reported by Ding and colleagues [3]. Our survey aims to identify potential side effects of COVID-19 vaccinations concerning menstrual irregularities among women across the globe. With this survey, we aim to identify a potentially relevant side effect for women of reproductive age trying to conceive, postmenopausal women who may consider breakthrough bleeding as a sign of serious disease, as well as transwomen. We also explore plausible mechanisms that may result in menstrual irregularities following COVID-19 vaccine.

## 2 Methods

A population-based cross-sectional study was conducted by means of an electronic survey distributed to healthcare workers and the general population members, who were female, across the world. Using online media platforms, the survey was distributed from June 10, 2021, to July 10, 2021. The Institutional Review Board (Ministerio de Salud Publica) (Ref No. 024–2020) at Universidad de Especialidades Espíritu Santo, Ecuador approved the study before the distribution of the survey. This study was additionally registered with Research Registry: researchregistry7876 [4].

The survey instrument was created to address the study objectives. This involved wording the questions based on the baseline characteristics (2 questions), vaccination status and dosage obtained (2 questions), menstruation and related factors (11 questions), thoughts and knowledge about menstrual irregularities (2 questions). Participants were asked their HCW status and whether they thought menstrual irregularities could have been caused due to the COVID-19 vaccine with six answer choices: strongly agree, agree, don't know, disagree, strongly disagree, and not applicable. They were also questioned concerning their knowledge about menstrual irregularities following COVID-19 vaccination, with two answer choices: yes, or no.

The target population was selected based on non-probability convenience sampling, comprising females worldwide who wished to voluntarily partake in the study. Sample size was calculated with OpenEpi software to be 384 using the estimate of population size to be 1,000,000 persons due to lack of exact number of population size being addressed. The sample size was calculated using the following formula: [DEFF*Np(1-p)]/[(d2/Z21-α/2*(N-1) +p*(1-p)]. The predicted hypothesis of outcome factor was estimated as 50% as there are no clear studies in the subject. The confidence interval was 95%, and accepted margin of error was 5%. All participants were informed of the survey details using a consent form to participate on beginning the survey, which was approved by the ethical review committee. The aim of the study along with the consent form was attached to the questionnaire. Those who did not wish to participate and responses that were incomplete were excluded. The STROCSS 2021 checklist is appended in the supplementary materials [5].

Questionnaire responses were analyzed using Statistical Package for the Social Sciences (SPSS v25). Descriptive statistics were summarized for baseline characteristics, vaccination and dosage, and factors associated to menstrual irregularities, and thoughts and knowledge about menstrual irregularities. Bivariate analyses were used to compare the characteristics of responses to knowledge about menstrual irregularities. Furthermore, the chi-square test of independence was conducted to determine associations between HCW status, perceptions, and knowledge of COVID-19 vaccination. A sensitivity analysis was conducted to omit confounders, which was presented separately in the results. This sub analysis only comprised of 340 vaccinated women with regular menstrual periods, who did not take any medications (i.e., OCPs or NSAIDS) that could impact periods in the last 12 months before getting vaccinated, and who had at least one or more menstrual periods post-vaccination.

The receiver operating characteristic (ROC) analysis was conducted to assess the sensitivity and specificity of all variables associated to knowledge about menstrual irregularities. Because of the anticipated biases subjected to participants in the questionnaire responses, the AUC value of over 0.5 was considered to be a good model.

## 3 Results

With 590 all-time clicks, 510 respondents complete the survey, giving rise to a response rate of 86.3%. On using the entire sample (N = 510), no association was found between the HCW status and thoughts about menstrual irregularities (χ2) = 10.422, p = 0.064. Similarly, we found that the HCW status and knowledge about menstrual irregularities following COVID-19 vaccination had no associations as well (χ2) = 1.966, p = 0.161.

To omit confounders, the chi-square test of independence was re-run by include 1) Vaccinated, 2) With regular menstrual periods, 3) who did not take any medications (i.e., OCPs or NSAIDS) that could impact periods in the last 12 months before getting vaccinated, 4) who had at least one or more menstrual periods post-vaccination; N = 340. The re-run yielded the following associations between vaccinated individuals with regular periods, who did not take any medications that could impact periods in the last one year, and who had at least one period post-vaccination to thoughts about menstrual irregularities (χ2) = 7.418, p = 0.191 and knowledge about menstrual irregularities (χ2) = 3.137, p = 0.077.

Of the 314 HCWs, 60 (19.1%) heard of menstrual irregularities following COVID-19 vaccination from friends, family or colleagues (OR = 1.338; 95 CI = 0.886–2.019). On the other hand, 28 (14.3%) of the general population (N = 196) heard of the irregularities from friends, family, or colleagues (OR = 0.944; 95% CI = 0.873–1.021). On asking the HCWs vs. the general population whether menstrual irregularities may be caused by the vaccine, 9 (2.9%) vs. 4 (2%) strongly agreed, 15 (4.8%) vs. 5 (2.6%) agreed, 82 (26.1%) vs. 69 (35.2%) did not know, 77 (24.5%) vs. 43 (21.9%) disagreed, 62 (19.7%) vs. 24 (12.2%) strongly disagreed, and 69 (22%) vs. 51 (26%) chose not applicable. Among all participants (N = 510), vaccinated individuals presented with a higher risk of change in length of the cycle between periods (OR = 3.172; 95% CI = 0.470–21.431), as opposed to the non-vaccinated respondents (OR = 0.864; 95% CI = 0.762–0.980) (see Table 1).

**Table 1:** Demographics, vaccination status, traits of menstruation, medications, perceptions, and knowledge about menstrual irregularities due to COVID-19 vaccine.

	N (%)	χ2	p-value (bivariate)
Healthcare worker		1.966	0.162
Yes	314 (61.6)		
No	196 (38.4)		
Country of residence		22.783	0.001
India	195 (38.2)		
Pakistan	178 (34.9)		
USA	67 (13.1)		
Bangladesh	18 (3.5)		
Nepal	13 (2.5)		
Canada	7 (1.4)		
Ecuador	6 (1.2)		
Cyprus	5 [1]		
UK	5 [1]		
Malaysia	2 (0.4)		
UAE	2 (0.4)		
Bolivia	1 (0.2)		
Dominica	1 (0.2)		
Germany	1 (0.2)		
Guyana	1 (0.2)		
Italy	1 (0.2)		
Japan	1 (0.2)		
KSA	1 (0.2)		
Kuwait	1 (0.2)		
Nigeria	1 (0.2)		
Oman	1 (0.2)		
Sint Maarten	1 (0.2)		
Venezuela	1 (0.2)		
Vaccinated		0.002	0.965
Yes	493 (96.7)		
No	17 (3.3)		
Doses received to complete the vaccination		1.923	0.443
Completed after one dose	49 (9.6)		
Completed after two doses	335 (65.7)		
Incomplete after one dose	126 (24.7)		
Age on first period		–	0.585
Age (Mean ± SD)	13.14 ± 2.768		
Periods the last 12 months		8.032	0.238
My periods have stopped	9 (1.8)		
They have been irregular for a few months	84 (16.5)		
They have never been regular	417 (81.8)		
Cause of irregular periods before being vaccinated		22·438	0·680
Currently breastfeeding	4 (0.8)		
Currently pregnant	9 (1.8)		
Dietary cause	9 (1.8)		
Environmental cause	7 (1.4)		
Exercise	1 (0.2)		
Physiological hormonal changes	11 (2.2)		
On treatment (e.g., hormonal IUD, contraceptive implants)	31 (6.1)		
Menopause	16 (3.1)		
Polycystic ovary syndrome	98 (19.2)		
Pre-menopause	2 (0.4)		
Stress	35 (6.9)		
Recent weight fluctuation	10 [2]		
Not applicable	277 (54.3)		
Usual interval between periods or the usual interval between periods before they became irregular or stopped		2.037	0.792
Less than 24 days	45 (8.8)		
24–26 days	100 (19.6)		
24–26 days	171 (33.5)		
30–32 days	142 (27.8)		
More than 35 days	52 (10.2)		
Change in the length of cycle between periods after getting vaccinated for COVID-19		66.913	<0.001
Yes	93 (18.2)		
No	417 (81.8)		
Periods sooner or later after getting vaccinated		40.143	0.024
Later	92 [18]		
Same	366 (71.8)		
Sooner	52 (10.2)		
Periods after being vaccinated		4.936	0.626
0	42 (8.2)		
1	175 (34.3)		
2	130 (25.5)		
3	85 (16.7)		
4	50 (9.8)		
5	23 (4.5)		
6	5 [1]		
Witnessed heavier or lighter bleeding during periods after getting vaccinated		17.365	<0.001
Heavier	50 (9.8)		
Lighter	74 (14.5)		
Same	386 (75.7)		
Bleeding for more or fewer days after getting vaccinated, as compared to periods in the last 12 months		22.922	<0.001
Fewer days	58 (11.4)		
More days	36 (7.1)		
Same	416 (81.6)		
Consumed any medications that could impact periods (e.g., OCPs or NSAIDs) in the last 12 months before being vaccinated		6.981	0.008
Yes	99 (19.4)		
No	411 (80.6)		
Consumed any medications to help with periods (e.g., OCPs or NSAIDS) after being vaccinated		0.577	0.449
Yes	90 (17.6)		
No	420 (82.4)		
Thoughts about menstrual irregularities possibly caused by the COVID-19 vaccine		91.924	<0.001
Strongly agree	13 (2.5)		
Agree	20 (3.9)		
Don't know	151 (29.6)		
Disagree	120 (23.5)		
Strongly disagree	86 (16.9)		
Not applicable	120 (23.5)		
Heard of menstrual irregularities following the COVID-19 vaccination from friends, family, or colleagues		reference	<0.001
Yes	88 (17.3)		
No	422 (82.7)		

**Fig. 1:**
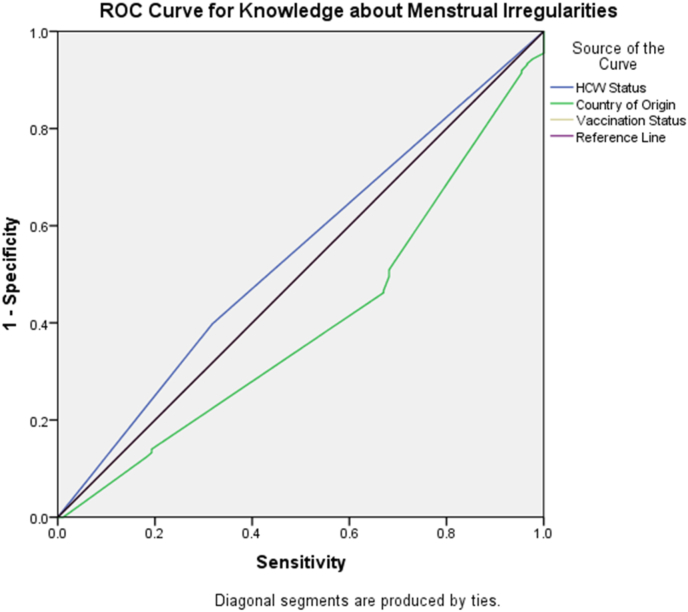
ROC curve for the sensitivity and specificity of knowledge about menstrual irregularities. The curve shows that the country of origin is a generally good model, with vaccinated individuals being more knowledgeable of menstrual irregularities. Knowledge about menstrual irregularities is the reference line.

**Fig. 2:**
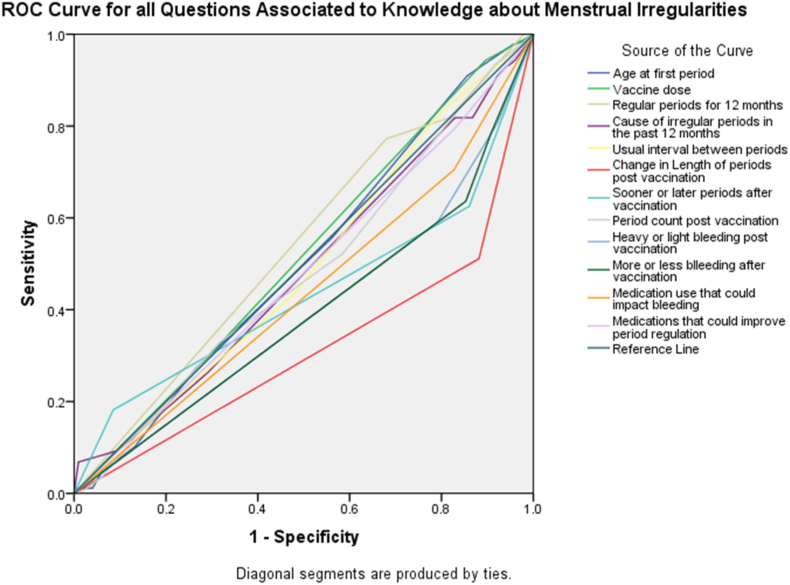
ROC curve for factors with the strongest association to knowledge about menstrual irregularities. Knowledge about menstrual irregularities is the reference line.

Fig. 1, Fig. 2 showcases findings from the sensitivity and specificity analysis using the ROC curve for all questions about the knowledge of menstrual irregularities due to COVID-19 vaccination. We found that the country of origin of the participant (AUC = 0.602, 95% CI = 0.538–0.666) had the strongest association to knowledge about menstrual irregularities, with vaccination status (AUC = 0.5, 95% CI = 0.434–0.567) being an acceptable model (Fig. 1).

The strongest associations to knowledge in the questionnaire were found with the vaccine dosage (AUC = 0.519, 95% CI = 0.455–0.585), regularity of periods in the previous 12 months (AUC = 0.54, 95% CI = 0.474–0.605), and age at first period (AUC = 0.508, 95% CI = 0.44–0.571) (Fig. 2).

## 4 Discussion

So far, there has been no link to changes in ovarian reserve following IVF among vaccinated women [6]. Menstrual changes may impact the ovulatory status of women who have not received stimulatory cycles. Potential ovulatory changes induced by vaccination are relevant for women of reproductive age who are trying to conceive [7]. For postmenopausal women and transwomen, the breakthrough bleeding may also represent signs of cancer [8,9]. There is a gap in the current understanding of menstrual irregularities, even if temporary, following COVID-19 vaccination that requires further exploration. Misinformation may also be the culprit for the observed proportion of women that noticed changes in their menstrual periods after COVID-19 vaccination. As reports of menstrual irregularities increase including breakthrough bleeding, missed or late periods, and heavy periods, it is important to consider the biological impact of COVID-19 vaccines.

There is an association between stress (e.g. physical and mental) and sex hormone fluctuations [10,11]. Observations regarding menstrual irregularities have been reported in literature following the HPV vaccine administration in young women [12]. There have been reports that have supported and opposed the possibility of associations between HPV vaccination and premature ovarian failure (POI) [13,14]. Possible underlying pathophysiological mechanisms are multifactorial, such as induced autoimmune responses and adjuvant adverse effects of vaccines [15]. These reports are relevant for certain COVID-19 vaccines, as aluminum salts have been used as an adjuvant in their development [16]. Both COVID-19 vaccines (e.g. Sinopharm) and HPV vaccines employ aluminum salt, used typically to enhance antigen-specific immune responses [17,18]. Not all COVID-19 vaccines contain the toxic ingredient (aluminum), however, some Chinese COVID-19 vaccines as well as vaccines used for other diseases do use tiny amounts of aluminum to boost the immune response [19]. Animal studies have demonstrated that aluminum may build up in the ovaries, impacting its integrity, weight, and ability to produce sex hormones. When observed in female rats, there has a suppression in the concentration of estrogen, progesterone, FSH, and LH resulting in abnormal development of follicles in the ovaries [20,21].

It is plausible that vaccine-induced thrombocytopenia is responsible for heavy menstrual bleeding associated with COVID-19 vaccines [22]. Reports of thrombosis with thrombocytopenia syndrome (TTS) following COVID-19 vaccination demonstrate thrombocytopenia of varying degrees, associated with thrombosis in unusual locations [23]. The underlying mechanism that is being considered is autoimmune heparin-induced thrombocytopenia (aHIT) [24]. Interestingly, TTS is more frequent in younger women aged 18–49 years [25]. It is plausible that menstrual irregularities may be compounded with thrombocytopenia and in rare cases, suggestive of TTS [22]. As efforts are being made to report vaccine adverse events to reporting systems, healthcare workers (HCWs) are advised to encourage and inform women to report these bleeding events or irregularities. From a public health perspective, these events may be temporary, however, they may indicate underlying severe adverse events such as TTS associated with a high rate of fatality.

## 5 Strengths and limitations

We reported findings of menstrual changes post covid-19 vaccination, of which evidence remains equivocal. In this study, we collected menstrual data from a large number of women; only the previous 3 months were inquired upon, thus we do not expect recall bias. Previous menstrual irregularities were also considered as well as baseline characteristics to control for confounders. The survey was anonymous which likely encouraged participants to respond despite the sensitive nature of the study. The findings of this study are robust due to the large sample size.

Our study has a few limitations. First, while we included an acceptable number of participants, we could not distribute paper forms, leading to reporting bias. Especially during the COVID-19 pandemic, with many non-peer reviewed scientific data such as media reports being used as information sources, self-reporting bias in responses may have positively or negatively tilted our findings. Second, as opposed to longitudinal study designs, cross-sectional survey study designs cannot account for clinical testing, thereby limiting causality. Third, there was no long-term follow up and this study presents a snapshot of survey findings about menstrual irregularities due to COVID-19 vaccine.

## 6 Conclusion

To summarize, our findings demonstrate changes in menstrual patterns after receiving one or more doses of the COVID-19 vaccine. The side effect has not been identified so far. This study is the first to explore menstrual irregularities among women across the globe following vaccination. It is pertinent to explain to women receiving the vaccination for COVID-19 that menstrual irregularities may be expected. For women who are focusing on conceiving or are post-menopausal, these menstrual irregularities are necessary to be aware of. Focused reproductive observatory safety studies are warranted to determine potentially short- and long-term adverse events of COVID-19 vaccines among women.

## Provenance and peer review

Not commissioned, externally peer-reviewed.

## Please state any conflicts of interest

None.

## Ethical approval

The Institutional Review Board (Ministerio de Salud Publica) (Ref No. 024–2020) at Universidad de Especialidades Espíritu Santo, Ecuador approved the study before the distribution of the survey.

## Please state any sources of funding for your research

None.

## Author contributions

Azza Sarfraz, Zouina Sarfraz: Conceptualization, Designing, Data Analysis, Writing (Original and Revision).

Muzna Sarfraz: Zainab Nadeem: Conceptualizing, Writing (Original and Revision), Data Interpretation.

Miguel Felix: Writing (Revision), Data Analysis and Interpretation.

Ivan Cherrez-Ojeda: Writing (Revision), Data Interpretation, Supervision, Guarantor.

## Registration of research studies

1. Name of the registry: Research Registry
2. Unique Identifying number or registration ID: researchregistry7876
3. Hyperlink to your specific registration (must be publicly accessible and will be checked): https://www.researchregistry.com/browse-the-registry#home/registrationdetails/6273fef870018f001e433e8c/

## Guarantor

Ivan Cherrez-Ojeda, Zouina Sarfraz.

## Consent

Participants were volunteers and required consent to partake in the survey. The declaration of Helsinki guidelines was followed throughout the course of this survey.
